# Utility of Smartphone-Based Digital Phenotyping Biomarkers in Assessing Treatment Response to Transcranial Magnetic Stimulation in Depression: Proof-of-Concept Study

**DOI:** 10.2196/40197

**Published:** 2023-09-01

**Authors:** Radhika Suneel Kelkar, Danielle Currey, Srilakshmi Nagendra, Urvakhsh Meherwan Mehta, Vanteemar S Sreeraj, John Torous, Jagadisha Thirthalli

**Affiliations:** 1 National Institute of Mental Health and Neurosciences Bangalore India; 2 Beth Israel Deaconess Medical Center Boston, MA United States

**Keywords:** theta burst stimulation, treatment response, predictive biomarker, outcome, digital phenotyping, transcranial magnetic stimulation, TMS, depression, smartphone, mobile phone

## Abstract

**Background:**

Identifying biomarkers of response to transcranial magnetic stimulation (TMS) in treatment-resistant depression is a priority for personalizing care. Clinical and neurobiological determinants of treatment response to TMS, while promising, have limited scalability. Therefore, evaluating novel, technologically driven, and potentially scalable biomarkers, such as digital phenotyping, is necessary.

**Objective:**

This study aimed to examine the potential of smartphone-based digital phenotyping and its feasibility as a predictive biomarker of treatment response to TMS in depression.

**Methods:**

We assessed the feasibility of digital phenotyping by examining the adherence and retention rates. We used smartphone data from passive sensors as well as active symptom surveys to determine treatment response in a naturalistic course of TMS treatment for treatment-resistant depression. We applied a scikit-learn logistic regression model (l1 ratio=0.5; 2-fold cross-validation) using both active and passive data. We analyzed related variance metrics throughout the entire treatment duration and on a weekly basis to predict responders and nonresponders to TMS, defined as ≥50% reduction in clinician-rated symptom severity from baseline.

**Results:**

The adherence rate was 89.47%, and the retention rate was 73%. The area under the curve for correct classification of TMS response ranged from 0.59 (passive data alone) to 0.911 (both passive and active data) for data collected throughout the treatment course. Importantly, a model using the average of all features (passive and active) for the first week had an area under the curve of 0.7375 in predicting responder status at the end of the treatment.

**Conclusions:**

The results of our study suggest that it is feasible to use digital phenotyping data to assess response to TMS in depression. Early changes in digital phenotyping biomarkers, such as predicting response from the first week of data, as shown in our results, may also help guide the treatment course.

## Introduction

Transcranial magnetic stimulation (TMS) is a noninvasive and safe method of selectively modulating aberrant brain circuits to drive therapeutic gains [1]. TMS has been in use as a treatment, typically administered as trains of repeated magnetic pulses for a few minutes a day, for 4-6 weeks. The most common clinical indications include major depression, obsessive-compulsive disorder, substance dependence, and schizophrenia [2]. Among these indications, the utility of TMS is primarily for the treatment of resistant or difficult-to-treat conditions that fail to respond to conventional therapies. Although there is strong evidence for the clinical benefits of TMS in the treatment of these disorders, TMS is most widely used in the treatment of resistant major depression. TMS first received regulatory approvals for the treatment of depressive disorders, and over the years, there has been a substantial pool of evidence that supports the use of different TMS therapies—conventional, patterned, and deep TMS—for treating depression [3]. Owing to the already persistent nature of symptoms, the response rates for major depression with TMS are variable, ranging from 30% to 60% [4]. Identifying biomarkers of response to TMS is a priority for personalizing care [5,6]. Clinical characteristics observed before starting TMS have not yet been consistent indicators of the prospective response to TMS [7]. Smartphone-based digital phenotyping biomarkers offer a promising and scalable means to characterize behavior across multiple domains, including symptomatic, physiological, and cognitive domains [8]. Digital phenotyping refers to “moment-by-moment quantification of the individual-level human phenotype in situ using data from smartphones and other personal digital devices” [9]. This is achieved using passively obtained data, such as accelerometer readings, geolocation information, and call or text logs. Smartphone apps that apply these approaches have been used in various psychiatric illnesses—particularly depression and schizophrenia—to monitor psychological, physiological, and behavioral measures. Studies using smartphone app–based digital phenotyping to monitor response to treatment have found that improvements in digital phenotyping–based parameters, such as sleep and cognition, occur before subjects actually perceive and report improvements in symptoms on assessment scales [10]. This is feasible, as smartphone digital phenotyping uses sensors in personal devices to transform metrics like real-time accelerometer data or real-time geo-location data into behavioral features like sleep duration or home time, respectively. Digital phenotyping smartphone apps can also facilitate actively obtained real-time surveys and cognitive assessments. A combination of such active and passive data captured within a defined time scale has been shown to have predictive utility in determining prospective clinically relevant outcomes [11]. The potential of such multimodal, scalable, and longitudinal monitoring has not been investigated to assess response to repetitive transcranial magnetic stimulation (rTMS) in patients diagnosed with major depression. The longitudinal predictive utility of such smartphone-derived digital phenotypes has recently been demonstrated in predicting relapse in schizophrenia across diverse sociocultural and geographical settings [11]. In this proof-of-concept study, we assessed the feasibility and clinical utility of smartphone-based digital phenotyping in predicting the response to TMS among individuals with a major depressive disorder recruited in a naturalistic clinical setting. Feasibility was assessed via adherence and retention rates. We hypothesized that more than 70% of patients would complete the study. Clinical utility was assessed using the accuracy of predicting response rates to TMS treatment. We hypothesized that the accuracy of digital phenotyping in predicting the response to TMS would be more than that achieved by chance (50%).

## Methods

### Study Design and Participants

The study was conducted as an open-label, single-arm feasibility trial. Participants were recruited based on a nonprobability convenience sampling after being referred to the brain stimulation center at a tertiary care hospital in southern India.

We included adult patients of either gender with a primary diagnosis of either unipolar or bipolar depression, according to the *International Classification of Diseases, Tenth Revision*, who had failed to respond to at least one adequate trial of an antidepressant [12]. They met the inclusion criteria if they were able to read and write in English and had access to a smartphone with an internet connection. Exclusion criteria included diagnoses of dysthymia, cyclothymia, or intellectual disability. We also excluded patients who could not use the proposed application due to reasons such as the unavailability of a smartphone, the presence of sensory abnormalities, or difficulties in comprehending the English language.

### Intervention

Patients received rTMS treatments administered with a MagVenture MagPro X100 device, involving sequential bilateral theta burst stimulation (TBS) over the dorsolateral prefrontal cortex manually localized 7 cm anterior to the motor hotspot. Each session comprised 1800 pulses each of intermittent TBS to the left dorsolateral prefrontal cortex and continuous TBS to the right dorsolateral prefrontal cortex, both delivered at 90% of the resting motor threshold. The rTMS treatments were administered once daily, 6 days a week, for a duration of 3 to 4 weeks [13].

Following the baseline clinical assessments, MindLAMP, a freely available smartphone-based app (compatible with both iOS and Android systems) was installed on the patients’ mobile phones; patients were then registered with a unique ID. MindLAMP collected both active and passive data. Active data consisted of symptom surveys and cognitive tasks (brain games) along with environment and context tagging. In passive data collection, the app collected various parameters, such as physical activity (total steps walked in 24 hours), relative physical position using a global positioning system (without information of precise locations), as well as phone use and screen use data.

### Assessments

The adherence was assessed by dividing the number of participants with available active data by the total number of participants who completed the study. The retention rate was calculated by dividing the total number of participants who completed the study by the total number of individuals recruited.

#### Digital Biomarkers

Digital phenotyping data included features derived from passively acquired accelerometer data (sleep duration), geolocation data (entropy, home time, and GPS data quality), screen state data (screen-on duration), and actively acquired symptom surveys—Patient Health Questionnaire (PHQ-9) and Generalized Anxiety Disorder Screener (GAD-7) [14,15]. Specifically, home time was estimated by pooling significant locations by the specified resolution to determine the amount of time an individual spent at home within that time window. Entropy was estimated as the variability of the time a participant spent at significant locations determined by their GPS data. Accelerometer data were set to be sampled at 5 Hz, and GPS was set to be sampled at 1 Hz, but actual data collection occurred at rates lower than these preset values. Screen capture data were read directly from the operating system. Symptom surveys were offered via the app every day. Together, these metrics yielded critical behavioral information that is often not available during in-person clinical interviews, and therefore, they served as potential novel markers of change or improvement in symptoms following treatment with TMS.

#### Clinical Assessments

Clinical symptom severity assessments using the Hamilton Depression Rating Scale (HDRS) [16] were performed every week by a trained psychiatrist prior to TMS treatment and at the end of the TMS treatment.

### Outcome Definition

Response to TMS was defined as a reduction of >50% in the HDRS score from baseline to the end of the treatment. Remission was estimated as an HDRS score <8 at the end of the TMS treatment.

### Data Analysis

Digital phenotyping features were calculated using the open-source cortex package designed to work with MindLAMP data [17]. Features were computed on a day-by-day basis, and days without data were excluded. To predict binary response to rTMS, we applied a scikit-learn logistic regression model (l1 ratio=0.5) using the aforementioned data and related variance metrics throughout the entire duration of the treatment and individual treatment weeks. We performed 2-fold cross-validation. The study was conducted between July 2021 and March 2022.

### Ethical Considerations

The National Institute of Mental Health and Neurosciences ethics committee approved the study protocol on June 4, 2020 (NIMH/Psy/DESC/BSP/2020/03). All data were deidentified, encrypted, and securely stored for analysis. All participants signed a written informed consent.

## Results

A total of 26 patients who met the inclusion criteria were screened. Of them, 23 provided consent for the study, and 19 completed the study. Patients received an average of 18 (SD 6) TBS sessions. After 2 more dropouts, a total of 17 patients completed the survey and provided passive and clinical follow-up data and were included in this analysis. These participants did not exhibit significant differences from the 6 participants who dropped out in terms of any baseline clinical characteristics (Table 1).

The adherence rate was calculated to be 89.47%, and the retention rate was 73%. GPS data coverage was computed as the percentage of 10-minute windows with at least one data point in the study and was on average 43%. The average percentage change on the HDRS was 41.4% (SD 36.2%); among the 17 participants, 8 achieved both response (defined as an HDRS reduction of ≥50% from baseline) and remission (defined as an HDRS score <8). No serious adverse effects were reported following TMS.

Given the pilot nature of the analyses, we explored different models that could determine the status of the treatment response to TMS. We report the area under the curve (AUC) for each of the models to enable the interpretation of model accuracy. The AUC for the correct classification of TMS response based on the average of all passive data features over the entire duration of the treatment was 0.625. To increase the size of the data set, we used the average of all features on a weekly basis (spanning a total of 46 weeks); the AUC for this model was 0.59. When including the variance of passive data features and individual survey questions (active features), the AUC was 0.911. We were also interested in the early prediction of treatment response in the study. A model using the average of all features (passive and active) for the first week yielded an AUC of 0.7375. In the best-performing model (AUC 0.911), digital phenotyping features derived from geolocation (home time and entropy) were the 2 nonzero passive data model coefficients. A full list of coefficients from this model can be found in Table 2.

**Table 1:** Clinical characteristics of patients recruited.

Characteristics			Overall (N=23)	Dropouts^a^ (n=6)		Complete data (n=17)		*P* value^b^	
Age (years), mean (SD)			35 (14)	39 (16)		34 (14)		.53	
**Gender, n (%)**									.64
	Female	11 (48)		2 (33)	9 (53)				
	Male	12 (52)		4 (67)	8 (47)				
Duration of illness (years), mean (SD)			10 (10)	9 (6)		11 (11)		.51	
**Depression type, n (%)**									.34
	Bipolar	10 (43)		4 (67)	6 (35)				
	Unipolar	13 (57)		2 (33)	11 (65)				
Duration of current episode (months), mean (SD)			13.0 (19.0)	10.2 (7.7)		14.0 (21.8)		.55	
Baseline HDRS^c^ (total), mean (SD)			21 (4)	20 (1)		21 (4)		.53	
RMT^d^ (left hemisphere), mean (SD)			37 (6)	36 (6)		37 (6)		.67	
RMT (right hemisphere), mean (SD)			38 (6)	38 (5)		38 (7)		.91	
Total TMS^e^ sessions, mean (SD)			18 (6)	15 (10)		19 (4)		.33	

^a^Regarding the dropouts, 2 participants discontinued treatment with transcranial magnetic stimulation because of worsening symptoms requiring electroconvulsive therapy, and 1 participant developed a seizure and discontinued; 3 could not use the app because of phone compatibility issues.

^b^Fisher exact test was used for categorical data, and independent 2-tailed *t* test was used for continuous data.

^c^HDRS: Hamilton Depression Rating Scale.

^d^RMT: resting motor threshold.

^e^TMS: transcranial magnetic stimulation.

**Table 2:** Nonzero model coefficients for the best-performing model (area under the curve 0.911), using all features and variances.

Feature	Coefficient
Home time	0.157
Entropy	–0.191
Today I felt little interest or pleasure.	–0.323
Today I feel depressed.	–0.051
Today I feel tired or have little energy.	–0.055
Today I have a poor appetite or am overeating.	–0.696
Today I have trouble focusing or concentrating.	–0.334
Today I feel anxious.	–0.373
Today I cannot stop worrying.	–0.094
Today I am easily annoyed or irritable.	–0.673
Today I have a poor appetite or am overeating (variance).	–0.301
Today I have thoughts of self-harm (variance).	–0.120
Today I am easily annoyed or irritable (variance).	0.005

## Discussion

### Principal Findings

To the best of our knowledge, this is the first study testing the feasibility of digital phenotyping in patients receiving rTMS for depression treatment. Based on the adherence and retention rates, it can be concluded that it is feasible to use digital phenotyping data to assess the response to TMS. Digital biomarkers have the potential for scalability and cost-effectiveness in addition to being sensitive to clinically relevant phenotyping across mood [18] and psychotic [11] disorders. The utility of supporting continuous behavioral measurements outside the constraints of the clinical environment makes digital biomarkers particularly attractive in supporting prognosis, symptom tracking, and overall improved clinical care [19]. Our observations regarding feasibility can expand the utility of digital technologies in aiding clinical decision-making throughout the course of TMS treatment for depressive disorders.

Although it is not surprising that the model with the most features performed the best, the model included a combination of the sensor (GPS) and survey data, which highlights the ability to capture relevant multimodal data on patient response. Classical predictors of response to TMS include prior treatment failures, comorbidities, and the duration of the current episode [13,20,21], but adding digital phenotyping predictors could be a useful complement. These digital phenotyping predictors could also be used to screen patients for more expensive or time-consuming predictors, such as electroencephalography or neuroimaging-derived markers. Early changes in digital phenotyping biomarkers, such as predicting response from the first week of data, as shown in our results, may also help guide the treatment course.

### Limitations and Conclusions

We acknowledge that due to the small study sample in this pilot research, the variability in the reported AUC scores will be high. Although we demonstrate the feasibility of app-based digital data collection in the clinical context of TMS for depression, our preliminary associations between smartphone digital phenotyping and response are only indicative of the potential future clinical promise this technique might hold. These findings can, therefore, enable us to conduct larger and well-powered studies to confirm the predictive potential of digital phenotyping in TMS response for depression. We could have captured other measures through the MindLAMP application, such as activity levels and other psychosocial features. However, given the exploratory nature of this pilot study, we restricted the assessments to the ones mentioned above. Future studies need to replicate these pilot observations in similar longitudinal studies with larger samples and across diverse clinical settings. The stability and longevity of the observed changes after the last TMS treatment also merit further investigation. In this study, we did not include cognitive, voice, or physiological smartphone-based biomarkers, which may offer further relevant data. Larger sample sizes will also be necessary to help identify and avoid overfitting as more biomarkers are explored. Although the retention rates might have been affected by other illness- and TMS-related factors as well, the adherence rates seem to be high due to the use of passive data collection for assessment. Thus, the utility of smartphone-based parameters is highly promising for a resource-limited country like India. Due to the scalability of smartphone digital phenotyping, with over 80% of the world population already owning a smartphone, larger studies are feasible. Methods to further increase the quality of digital phenotyping data will also increase confidence in the derived features. As the field of digital phenotyping matures, new study procedures and data quality checks can help ensure that the sensor data are captured with the highest coverage possible.

## Abbreviations

AUC: area under the curve
GAD-7: Generalized Anxiety Disorder Screener-7
HDRS: Hamilton Depression Rating Scale
PHQ-9: Patient Health Questionnaire-9
rTMS: repetitive transcranial magnetic stimulation
TBS: theta burst stimulation
TMS: transcranial magnetic stimulation
